# Prognostic analysis and development of a prognostic model for EML4-ALK-positive lung cancer patients treated with ALK-TKIs

**DOI:** 10.3389/fmed.2026.1760300

**Published:** 2026-04-07

**Authors:** Ruimin Liu, Chunrong Su, Hang Su, Lisen Wang

**Affiliations:** 1 Oncology Department of Integrated Traditional Chinese and Western Medicine, Zhumadian Central Hospital (Zhumadian Central Hospital Affiliated to Huanghuai College), Zhumadian, Henan, China; 2 The Second Department of Oncology, Zhumadian Central Hospital (Zhumadian Central Hospital Affiliated to Huanghuai College), Zhumadian, Henan, China

**Keywords:** ALK-TKIs, EML4-ALK, lung cancer, prognosis, prognostic model

## Abstract

**Objective:**

This study aimed to investigate prognostic factors and establish prognostic models for EML4-ALK-positive non-small cell lung cancer (NSCLC) patients receiving ALK tyrosine kinase inhibitors (TKIs) treatment.

**Methods:**

We retrospectively analyzed 114 ALK-positive NSCLC patients treated at our institution from January 2020 to January 2024. Clinical data, laboratory results, imaging findings, and follow-up records were collected. Progression-free survival (PFS) and overall survival (OS) served as primary endpoints. Statistical methods included Kaplan–Meier analysis, Cox regression modeling, and nomogram construction (70% training set, 30% validation cohort). Model performance was evaluated using Harrell’s C-index, calibration curves, and time-dependent receiver operating characteristic (ROC) analysis.

**Results:**

The median PFS was 30.97 months (95% CI: 25.43–36.52), while median OS was not reached. Multivariate analysis identified four independent PFS predictors: brain metastasis (HR = 2.15, *p* = 0.008), central lesion location (HR = 1.89, *p* = 0.013), tumor diameter >3 cm (HR = 1.76, *p* = 0.022), and lymphocyte-to-monocyte ratio ≤2.26 (HR = 1.92, *p* = 0.011). For OS, significant factors included brain metastasis (HR = 2.87, *p* = 0.002), CYFRA21-1 level (HR = 1.65, *p* = 0.028), and LMR (HR = 2.04, *p* = 0.007). The developed nomograms demonstrated good prognostic accuracy, with validation cohort C-indices of 0.73 (PFS) and 0.75 (OS). Time-dependent AUCs for 3-year PFS and 4-year OS prognostic were 0.79 and 0.78, respectively.

**Conclusion:**

Our study established that brain metastasis status, tumor characteristics, and systemic inflammatory markers significantly impact clinical outcomes in ALK-positive NSCLC patients undergoing TKI therapy. The developed prognostic models show satisfactory prognostic performance and may assist in clinical decision-making and patient stratification. These findings warrant further validation in prospective, multicenter studies.

## Introduction

Non-small cell lung cancer (NSCLC) is the most common type of lung cancer in clinical practice, accounting for approximately 85% of cases (1). Among NSCLC patients, approximately 3–7% harbor the echinoderm microtubule-associated protein like 4 (EML4)-anaplastic lymphoma kinase (ALK) fusion gene, which exhibits potent oncogenic activity and drives cellular transformation (2). With advancements in molecular targeted therapy for cancers, multiple ALK tyrosine kinase inhibitors (ALK-TKIs) have been progressively applied to patients with EML4-ALK-positive NSCLC, significantly improving their survival prognosis compared to traditional chemotherapy (3, 4). However, due to differences in genetic backgrounds and physical conditions, individual responses to ALK-TKI treatment vary. Therefore, identifying reliable biomarkers for predicting treatment response and prognosis is crucial for risk stratification and personalized therapy in these patients. While numerous studies have reported potential prognostic factors and biomarkers for NSCLC patients receiving chemotherapy or immunotherapy (5, 6), there is limited research on the prognostic factors specific to EML4-ALK-positive NSCLC treated with ALK-TKIs. In this study, we integrated clinicopathological features, molecular markers, and treatment-related variables to systematically analyze the prognosis of EML4-ALK-positive NSCLC patients treated with ALK-TKIs, identify prognostic factors, and develop an individualized prognostic prognostic model.

## Materials and methods

### General information

This was a retrospective study involving 114 patients with EML4-ALK-positive NSCLC who were treated with ALK-TKIs at Zhumadian Central Hospital Affiliated to Huanghuai College between January 2020 and January 2024. The study was approved by the Institutional Review Board (IRB) of Zhumadian Central Hospital Affiliated to Huanghuai College.

### Inclusion and exclusion criteria

Inclusion criteria: ① Aged ≥ 18 years old; ② diagnosed with NSCLC through pathological tissue examination, and the next-generation sequencing (NGS) test results show positive for the EML4-ALK fusion gene; ③ complete clinical and laboratory examination data, imaging data, and follow-up data are available; ④ the follow-up period is ≥ 12 months; ⑤ patients with stage IIIB-IV or locally advanced NSCLC who are unable to undergo radical surgery or radiotherapy and have received ALK-TKIs treatment; ⑥ the expected survival time is > 3 months (combined with the patients’ baseline performance status, tumor burden, laboratory findings, and clinical practice guidelines); ⑦ the Eastern Cooperative Oncology Group (ECOG) performance status score is 0–2 points; ⑧ no severe underlying diseases, severe cardiovascular and cerebrovascular diseases: New York Heart Association (NYHA) functional class III–IV, acute myocardial infarction within the recent 6 months, severe arrhythmia requiring long-term bed rest or device-assisted treatment, severe cerebral hemorrhage/cerebral infarction with severe limb dysfunction or disturbance of consciousness; severe hepatic and renal diseases: Child-Pugh grade C liver function, chronic renal failure (estimated glomerular filtration rate [eGFR] < 30 mL·min^−1^·1.73 m^−2^) or long-term hemodialysis/peritoneal dialysis; severe respiratory diseases: acute exacerbation of chronic obstructive pulmonary disease, severe asthma, moderate-to-severe interstitial lung disease requiring long-term oxygen therapy or mechanical ventilation support; other severe underlying diseases: severe coagulopathy (prothrombin time prolonged > 1.5 times the upper limit of normal with bleeding tendency), uncontrolled severe diabetes mellitus (fasting blood glucose > 16.7 mmol/L or recurrent diabetic ketoacidosis), history of other malignant tumors (other than the current EML4-ALK-positive NSCLC, other malignancies diagnosed within the past 5 years, excluding indolent tumors with favorable prognosis such as basal cell carcinoma of the skin and cervical carcinoma *in situ*); ⑨ there is at least one measurable tumor lesion.

Exclusion criteria: ① Patients with a history of other tumors; ② patients with other severe internal diseases and mental illnesses; ③ patients with poor compliance to follow-up and treatment; ④ patients with severe impairment of liver and kidney function; ⑤ patients with a history of long-term hormone treatment; ⑥ patients who received first-line treatment with lorlatinib (patients treated with lorlatinib are excluded because the drug has been on the market for a short time, the follow-up period is relatively short, and there is limited clinical data).

### Treatment programs

All patients received ALK-tyrosine kinase inhibitors (ALK-TKIs), including first-generation crizotinib and second-generation ceritinib, alectinib, ensartinib, and brigatinib. Among them, 32 patients received crizotinib, 47 received alectinib, 12 received ceritinib, 19 received ensartinib, and 9 received brigatinib. All agents were administered at standard clinical dosages, and treatment was continued until disease progression, death, or intolerable adverse reactions.

### Information collection

In this study, the clinical characteristics and laboratory test data of the patients were retrospectively collected through the hospital medical record system, mainly covering the following aspects:

1. Demographic data: Gender, age, body mass index (BMI), smoking history (a smoking index of ≥ 400 cigarettes/year is defined as having a smoking history);
2. Disease and treatment data: Pathological type (adenocarcinoma/non-adenocarcinoma), clinical TNM stage, presence of brain metastasis, presence of bone metastasis, EML4-ALK fusion subtype, previous treatment status, lesion location [central (tumors located adjacent to the lobar or segmental bronchi)/peripheral (tumors distal to the segmental bronchi)], maximum tumor diameter, Eastern Cooperative Oncology Group (ECOG) Performance Status (PS) score, type of tyrosine kinase inhibitor (TKIs);
3. Laboratory and inflammatory nutritional indicators: Serum carcinoembryonic antigen (CEA), cytokeratin 19 fragment (CYFRA21-1), neutrophils (NEUT), lymphocytes (LYM), Absolute Monocyte Count (AMC), serum albumin (ALB), and calculation of the neutrophil-to-lymphocyte ratio (NLR), lymphocyte-to-monocyte ratio (LMR), and prognostic nutritional index (PNI). NLR = NEUT/LYM, LMR = LYM/AMC, PNI = ALB (g/dL) + 5 × LYM (10^9^/L).

### Follow-up and outcome indicators

For EML4-ALK positive NSCLC patients, after the initiation of ALK-TKIs treatment, follow-up will be carried out every 2 to 3 months through outpatient reexamination (imaging + laboratory evaluation), standardized telephone follow-up, etc., and the follow-up period is ≥ 1 year. The follow-up endpoints are as follows: (1) death; (2) loss to follow-up, but the follow-up period has reached 12 months; (3) as of March 31, 2025. According to the follow-up situation, the outcome indicators, namely overall survival (OS) and progression-free survival (PFS), will be evaluated. OS refers to the time from the date of initiating treatment for the tumor to death (for any reason) or the last follow-up; PFS refers to the survival time from the date of initiating treatment for the tumor to disease progression or death.

### Statistical methods

Statistical analysis was performed using the SPSS 23.0 statistical software. The Kaplan–Meier method was used for univariate survival analysis, and the Log-rank method was used for significance testing. Univariate and multivariate Cox regression analyses were adopted to screen the influencing factors of OS and PFS. Based on the influencing factors screened by the multivariate Cox regression analysis, the R software was used to run the code to extract 70% of the data as the training cohort for constructing the nomogram prognostic model, and the remaining 30% of the data served as the validation cohort. The area under the receiver operating characteristic curve (AUC) and the Bootstrap method were used for internal data sampling verification to evaluate the value of the model. A *p*-value less than 0.05 was considered to indicate a statistically significant difference.

### Sample size calculation

According to Peduzzi’s rule, the sample size of this study was estimated using the formula *N* = 10 × the number of variables to be included/the event rate. Referring to the results of previous studies, the incidence of disease progression within 3 years among EML4-ALK positive NSCLC patients treated with ALK-TKIs was approximately 60% (7). In this study, a Cox regression model was used, and up to 6 covariates were included for multivariate analysis. By substituting the relevant parameters into the formula, the minimum required sample size was calculated to be 100 cases. Considering a dropout rate of 10%, it was finally determined that at least 110 subjects should be included. In this study, 114 patients were actually included, and the sample size met the basic statistical requirements, which could provide effective test efficiency for the research hypothesis.

## Result

As of March 31, 2025, the median follow-up time of this study was 29.77 months. Among the included patients, 63 cases (55.26%) experienced disease progression, and 51 cases (44.74%) did not. The median PFS was 30.970 months (95% CI, 25.425–36.515); 83 patients (72.81%) survived, and the median OS was not reached.

Age, BMI, NLR, LMR, PNI, CEA, and CYFRA21-1 were grouped according to the median value. The results of Kaplan–Meier univariate survival analysis showed that patients with brain metastasis, central location of the lesion, maximum tumor diameter > 3 cm, LMR ≤ 2.26, and first-generation tyrosine kinase inhibitors (TKIs) had a shorter PFS; patients with brain metastasis, LMR ≤ 2.26, PNI ≤ 45.95, and CYFRA21-1 > 8 μg/mL had a shorter OS; all with *p* < 0.05. See Table 1 for details.

**Table 1:** Relationship between clinical characteristics of patients and prognosis.

Clinical information	*n*	median PFS (month)	*X* ^2^	*p*-value	Median OS (month)	*X* ^2^	*p*-value
Gender			0.718	0.397		0.021	0.883
Male	45	26.63 (18.95–34.32)			Not achieved		
Female	69	34.40 (22.11–46.70)			Not achieved		
Age			2.770	0.096		0.037	0.847
≤51 year	57	43.23 (24.89–61.57)			Not achieved		
>51 year	57	27.13 (20.68–33.58)			Not achieved		
BMI			0.843	0.359		1.068	0.301
≤23.66 kg/m^2^	58	34.40 (22.14–46.66)			Not achieved		
>23.66 kg/m^2^	56	29.70 (18.10–41.30)			Not achieved		
Smoking history			0.018	0.893		0.349	0.555
No	84	31.23 (24.86–37.60)			Not achieved		
Yes	30	29.70 (19.39–40.01)			Not achieved		
Pathological type			0.004	0.948		0.035	0.852
Adenocarcinoma	107	31.23 (25.26–37.20)			Not achieved		
Non-adenocarcinoma	7	27.13 (23.09–38.85)			Not achieved		
TNM stage			1.591	0.451		0.530	0.767
IIIA	3	-			-		
IIIB	30	30.97 (22.08–39.86)			Not achieved		
IV	81	29.70 (20.21–39.19)			Not achieved		
Brain metastasis			6.569	0.010		7.158	0.007
No	74	33.40 (23.88–42.93)			Not achieved		
Yes	40	16.37 (12.47–20.27)			Not achieved		
Bone metastasis			1.371	0.242		3.231	0.072
No	79	32.10 (26.56–37.65)			Not achieved		
Yes	35	22.13 (6.38–37.88)			Not achieved		
EML4-ALK fusion subtype			5.107	0.164		5.184	0.159
V1	41	24.23 (8.41–40.06)			Not achieved		
V2	18	43.23 (35.85–50.61)			Not achieved		
V3a/b	50	30.97 (26.90–35.05)			Not achieved		
Other	5	17.37 (10.35–24.39)			Not achieved		
Lesion location			4.046	0.044		1.722	0.189
Central	66	41.33 (27.79–54.88)			Not achieved		
Peripheral	48	26.63 (20.16–33.10)			Not achieved		
Maximum tumor diameter			4.253	0.039		3.032	0.082
≤3 cm	48	34.40 (20.39–48.41)			Not achieved		
>3 cm	66	27.13 (21.71–32.55)			Not achieved		
ECOG PS score			1.550	0.461		0.727	0.695
0 points	18	34.40 (22.71–46.09)			Not achieved		
1 points	93	29.70 (22.00–37.40)			Not achieved		
2 points	3	23.90 (0.00–48.11)			Not achieved		
NLR			0.524	0.469		0.104	0.747
≤3.10	58	32.10 (24.67–39.54)			Not achieved		
>3.10	56	30.97 (20.03–41.91)			Not achieved		
LMR			4.138	0.043		6.650	0.010
≤2.26	60	23.63 (9.17–38.09)			Not achieved		
>2.26	54	39.27 (25.75–52.79)			Not achieved		
PNI			1.648	0.199		5.027	0.025
≤45.95	57	24.63 (12.97–34.30)			Not achieved		
>45.95	57	33.13 (23.14–45.66)			Not achieved		
CEA			1.024	0.312		2.163	0.141
≤15 ug/mL	60	33.13 (29.12–37.14)			Not achieved		
>15 ug/mL	54	23.63 (11.52–35.75)			Not achieved		
CYFRA21-1			3.490	0.062		5.421	0.020
≤8 ug/mL	58	34.40 (8.62–60.19)			Not achieved		
>8 ug/mL	56	28.80 (21.04–36.59)			Not achieved		
Previous treatment with platinum-based regimens			0.943	0.332		0.021	0.886
No	66	33.13 (15.15–51.11)			Not achieved		
Yes	48	29.70 (24.17–35.23)			Not achieved		
Type of TKIs			9.731	0.002		3.813	0.051
One generation	17	13.07 (7.96–18.18)			Not achieved		
Second generation	97	33.13 (24.12–42.14)			Not achieved		

BMI, body mass index; TKIs, tyrosine kinase inhibitors; NLR, neutrophil-lymphocyte ratio; LMR, lymphocyte-monocyte ratio; PNI, prognostic nutritional index; CEA, serum carcinoembryonic antigen; CYFRA21-1, cytokeratin 19 fragment.

Results of univariate and multivariate Cox regression analyses of factors affecting PFS in EML4-ALK positive lung cancer patients treated with ALK-TKIs.

The results of univariate Cox regression analysis showed that brain metastasis, EML4-ALK fusion subtype, lesion location, maximum tumor diameter, LMR, and the type of ALK-TKIs were all associated with the PFS of EML4-ALK positive lung cancer patients treated with ALK-TKIs (*p* < 0.05), as shown in Table 2. The statistically significant indicators from the univariate analysis were included in the multivariate Cox regression analysis. The results showed that brain metastasis, lesion location (central type), and maximum tumor diameter (>3 cm) were risk factors affecting PFS, and LMR (>2.26) was a protective factor affecting PFS; details are shown in Table 3. The model with the minimum AIC value (ΔAIC = 0) was defined as the optimal model. The optimal model determined by AIC-based sensitivity analysis was consistent with the main multivariate Cox regression results, indicating the stability of the variable selection (Table 4).

**Table 2:** Results of Cox univariate regression analysis of PFS.

Variable	*HR*	95%CI	*p*-value
Gender (male)	1.239	0.754–2.038	0.398
Age > 51 years old	1.532	0.923–2.543	0.099
BMI > 23.66 kg/m^2^	1.261	0.768–2.072	0.360
Smoking history (yes)	1.038	0.601–1.795	0.893
Pathological type (non-adenocarcinoma)	0.967	0.351–2.664	0.967
TNM staging (IV)	1.040	0.589–1.837	0.891
Brain metastasis (yes)	1.917	1.155–3.182	0.012
Bone metastasis (yes)	1.363	0.810–2.292	0.244
EML4-ALK fusion subtype	—	—	0.187
EML4-ALK fusion subtype (V2)	0.400	0.164–0.972	0.043
EML4-ALK fusion subtype (V3a/b)	0.883	0.517–1.507	0.647
EML4-ALK fusion subtype (others)	1.407	0.421–4.702	0.579
Lesion location (central type)	1.685	1.008–2.816	0.047
maximum tumor diameter (>3 cm)	1.718	1.021–2.891	0.042
ECOG PS score	—	—	0.468
ECOG PS score (1 point)	1.535	0.696–3.387	0.288
ECOG PS score (2 point)	2.285	0.473–11.038	0.304
NLR (>3.10)	1.202	0.730–1.978	0.470
LMR (>2.26)	0.590	0.352–0.987	0.044
PNI (>45.95)	0.687	0.418–1.129	0.138
CEA (>15 ug/mL)	1.291	0.786–2.120	0.313
CYFRA21-1 (>8 ug/mL)	1.609	0.972–2.662	0.064
Previous platinum regimen treatment (yes)	1.278	0.778–2.098	0.333
Types of TKIs (second generation)	0.355	0.187–0.674	0.002

BMI, body mass index; TKIs, tyrosine kinase inhibitors; NLR, neutrophil-lymphocyte ratio; LMR, lymphocyte-monocyte ratio; PNI, prognostic nutritional index; CEA, serum carcinoembryonic antigen; CYFRA21-1, cytokeratin 19 fragment.

**Table 3:** Results of multivariate Cox regression analysis of PFS.

Variable	*β*-value	SE-value	Wald-value	*p*-value	HR-value (95%Cl)
Brain metastasis	0.619	0.274	5.115	0.024	1.857 (1.086–3.175)
EML4-ALK fusion subtype (V2)	−0.607	0.466	1.700	0.192	0.545 (0.219–1.358)
Lesion location (central type)	0.686	0.288	5.685	0.017	1.987 (1.130–3.493)
maximum tumor diameter (>3 cm)	0.656	0.293	5.006	0.025	1.928 (1.085–3.426)
LMR (>2.26)	−0.748	0.285	6.910	0.009	0.473 (0.271–0.827)
Types of TKIs (second generation)	−0.479	0.324	2.184	0.139	0.619 (0.328–1.169)

LMR, lymphocyte-monocyte ratio.

**Table 4:** Results of AIC-based sensitivity analysis for PFS multivariate Cox regression model.

Model	Included variables	AIC value	ΔAIC	C-index (95%CI)
1 (full model)	Age > 51 years old, brain metastasis, EML4-ALK fusion subtype (V2), lesion location (central type), maximum tumor diameter (>3 cm), LMR (>2.26), CYFRA21-1 (>8 μg/mL), types of TKIs (second generation)	428.36	12.89	0.71 (0.63–0.79)
2	Brain metastasis, EML4-ALK fusion subtype (V2), lesion location (central type), maximum tumor diameter (>3 cm), LMR (>2.26), types of TKIs (second generation)	420.15	4.68	0.72 (0.64–0.80)
3	Brain metastasis, lesion location (central type), maximum tumor diameter (>3 cm), LMR (>2.26), types of TKIs (second generation)	417.82	2.35	0.72 (0.64–0.80)
4 (optimal model)	Brain metastasis, lesion location (central type), maximum tumor diameter (>3 cm), LMR (>2.26)	415.47	0.00	0.73 (0.65–0.81)
5	Brain metastasis, lesion location (central type), LMR (>2.26)	418.93	3.46	0.70 (0.62–0.78)
6	Brain metastasis, maximum tumor diameter (>3 cm), LMR (>2.26)	419.55	4.08	0.70 (0.62–0.78)

AIC, Akaike information criterion; ΔAIC, the difference between the AIC value of each model and the minimum AIC value; LMR, lymphocyte-monocyte ratio; TKIs, tyrosine kinase inhibitors.

### Univariate and multivariate Cox regression analysis results affecting OS in EML4-ALK-positive lung cancer patients treated with ALK-TKIs

The results of the univariate Cox regression analysis showed that brain metastasis, LMR, PNI, and CYFRA21-1 were all associated with the OS of EML4-ALK positive lung cancer patients treated with ALK-TKIs (*p* < 0.05), as shown in Table 5. The statistically significant indicators from the univariate analysis were included in the multivariate Cox regression analysis. The results showed that brain metastasis and CYFRA21-1 (>8 μg/mL) were risk factors affecting OS, and LMR (>2.26) was a protective factor affecting OS; details are shown in Table 6. The model with the minimum AIC value (ΔAIC = 0) was defined as the optimal model. The optimal model determined by AIC-based sensitivity analysis (Brain metastasis, LMR, CYFRA21-1) was consistent with the main results of multivariate Cox regression, indicating the stability of variable selection for the OS model. Considering the limited number of OS endpoint events, this result needs to be further verified with long-term follow-up (Table 7).

**Table 5:** Results of Cox univariate regression analysis of OS.

Variable	*HR*	95%CI	*p*-value
Gender (male)	1.055	0.517–2.154	0.883
Age > 51 years old	1.072	0.529–2.171	0.847
BMI > 23.66 kg/m^2^	1.450	0.714–2.944	0.304
Smoking history (yes)	1.288	0.555–2.990	0.556
Pathological type (non-adenocarcinoma)	0.872	0.208–3.659	0.852
TNM staging (IV)	1.066	0.503–2.263	0.867
Brain metastasis (yes)	2.538	1.252–5.144	0.010
Bone metastasis (yes)	1.904	0.932–3.891	0.077
EML4-ALK fusion subtype	—	—	0.263
EML4-ALK fusion subtype (V2)	0.133	0.018–1.005	0.051
EML4-ALK fusion subtype (V3a/b)	0.761	0.367–1.577	0.462
EML4-ALK fusion subtype (others)	1.134	0.148–8.689	0.904
Lesion location (central type)	1.607	0.786–3.285	0.193
maximum tumor diameter (>3 cm)	1.968	0.905–4.280	0.087
ECOG PS score	—	—	0.700
ECOG PS score (1 point)	1.613	0.486–5.353	0.435
ECOG PS score (2 point)	2.115	0.220–20.357	0.517
NLR (>3.10)	1.123	0.555–2.272	0.747
LMR (>2.26)	0.362	0.162–0.811	0.013
PNI (>45.95)	0.440	0.210–0.920	0.029
CEA (>15 ug/mL)	1.699	0.831–3.472	0.146
CYFRA21-1 (>8 ug/mL)	2.388	1.122–5.085	0.024
Previous platinum regimen treatment (yes)	1.054	0.516–2.152	0.886
Types of TKIs (second generation)	0.440	0.189–1.026	0.057

BMI, body mass index; TKIs, tyrosine kinase inhibitors; NLR, neutrophil-lymphocyte ratio; LMR, lymphocyte-monocyte ratio; PNI, prognostic nutritional index; CEA, serum carcinoembryonic antigen; CYFRA21-1, cytokeratin 19 fragment.

**Table 6:** Results of multivariate Cox regression analysis of OS.

Variable	*β*-value	SE-value	Wald-value	*p*-value	HR-value (95%Cl)
Brain metastasis	0.999	0.369	7.329	0.007	2.716 (1.318–5.599)
LMR (>2.26)	−0.938	0.423	4.918	0.027	0.391 (0.171–0.897)
PNI (>45.95)	−0.499	0.389	1.647	0.199	0.607 (0.284–1.301)
CYFRA21-1 (>8 ug/mL)	0.81	0.387	4.379	0.036	2.247 (1.053–4.789)

LMR, lymphocyte-monocyte ratio; PNI, prognostic nutritional index; CYFRA21-1, cytokeratin 19 fragment.

**Table 7:** Results of AIC-based sensitivity analysis for OS multivariate Cox regression model.

Model	Included variables	AIC value	ΔAIC	C-index (95%CI)
1 (full model)	Brain metastasis, bone metastasis, EML4-ALK fusion subtype (V2), maximum tumor diameter (>3 cm), LMR (>2.26), PNI (>45.95), CYFRA21-1 (>8 μg/mL), types of TKIs (second generation)	386.72	11.58	0.72 (0.63–0.81)
2	Brain metastasis, EML4-ALK fusion subtype (V2), Maximum tumor diameter (>3 cm), LMR (>2.26), PNI (>45.95), CYFRA21-1 (>8 μg/mL), types of TKIs (second generation)	379.25	4.11	0.73 (0.64–0.82)
3	Brain metastasis, maximum tumor diameter (>3 cm), LMR (>2.26), PNI (>45.95), CYFRA21-1 (>8 μg/mL)	377.53	2.39	0.73 (0.64–0.82)
4 (optimal model)	Brain metastasis, LMR (>2.26), CYFRA21-1 (>8 μg/mL)	375.14	0.00	0.75 (0.66–0.84)
5	Brain metastasis, LMR (>2.26)	378.86	3.72	0.72 (0.63–0.81)
6	Brain metastasis, CYFRA21-1 (>8 μg/mL)	379.42	4.28	0.71 (0.62–0.80)

AIC, Akaike information criterion; ΔAIC, the difference between the AIC value of each model and the minimum AIC value; LMR, lymphocyte-monocyte ratio; PNI, prognostic nutritional index; CYFRA21-1, cytokeratin 19 fragment; TKIs, tyrosine kinase inhibitors.

### Construction and efficiency analysis of nomogram models

#### PFS

**Figure 1:**
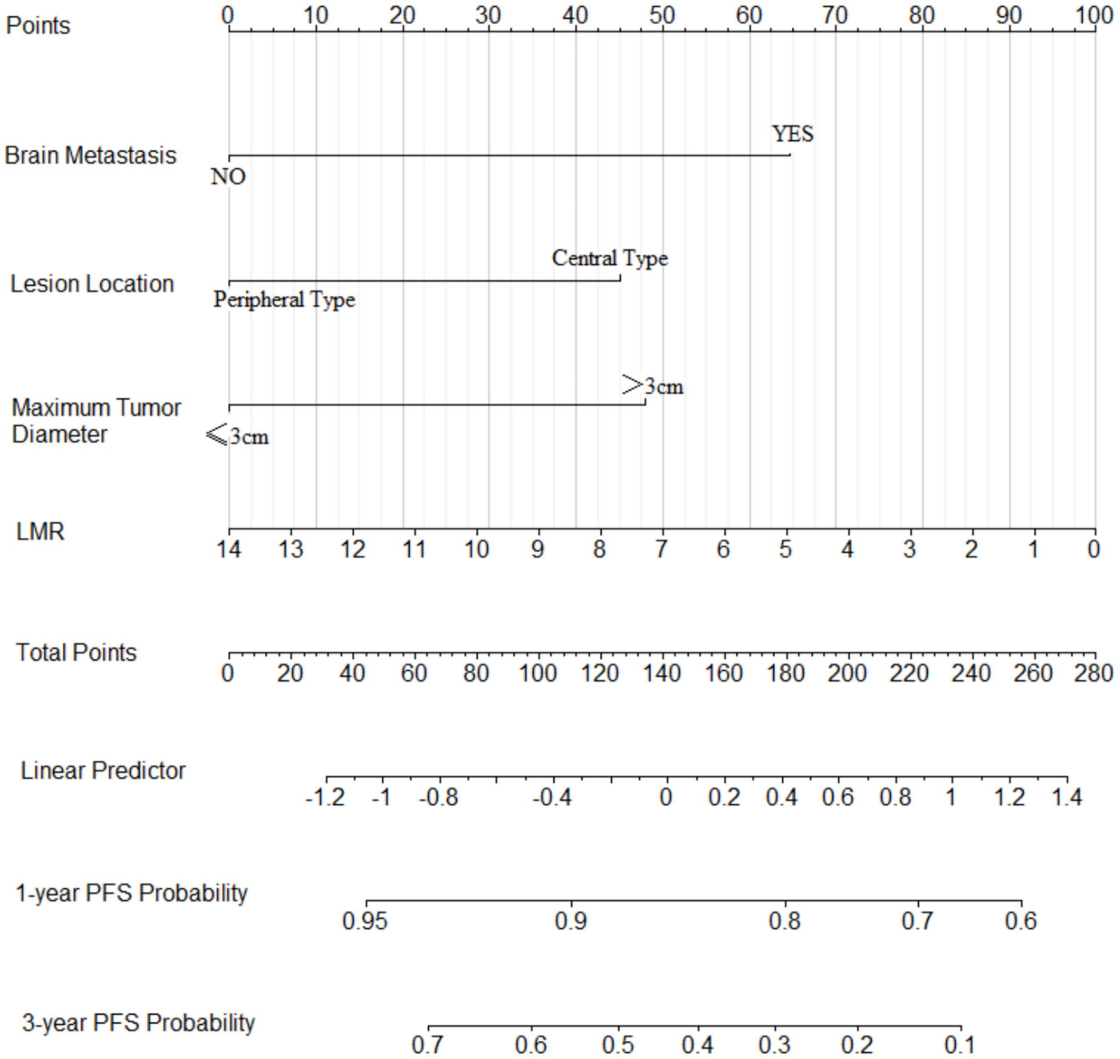
Nomogram model of the PFS rate. [Based on the four risk indicators (brain metastasis, lesion location, maximum tumor diameter, and LMR) screened by the multivariate Cox regression analysis, we constructed a nomogram model.]

**Figure 2:**
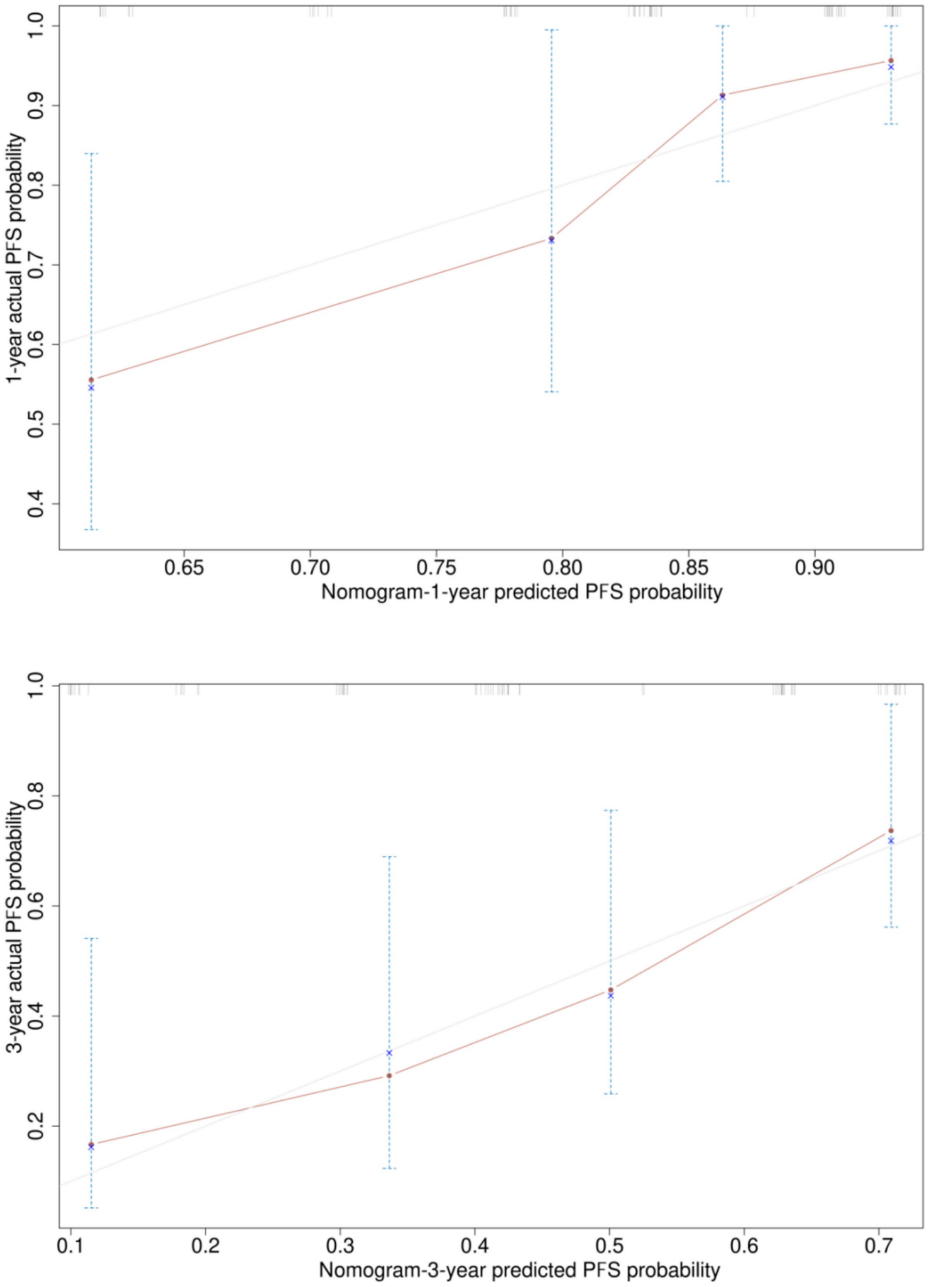
Calibration curve of the PFS rate training cohort. [Bootstrap resampling shows that the predicted probabilities of 1-year and 3-year PFS in the training cohort. The optimism-adjusted C-index after Bootstrap resampling (1,000 repetitions) was 0.71 (95% CI, 0.63–0.79), which corrected the overestimation of the original C-index (0.73, 95% CI, 0.65–0.81) caused by overfitting. This result indicates that the PFS prognostic model has good discriminative ability after adjusting for optimism, and the model performance is stable and reliable.]

**Figure 3:**
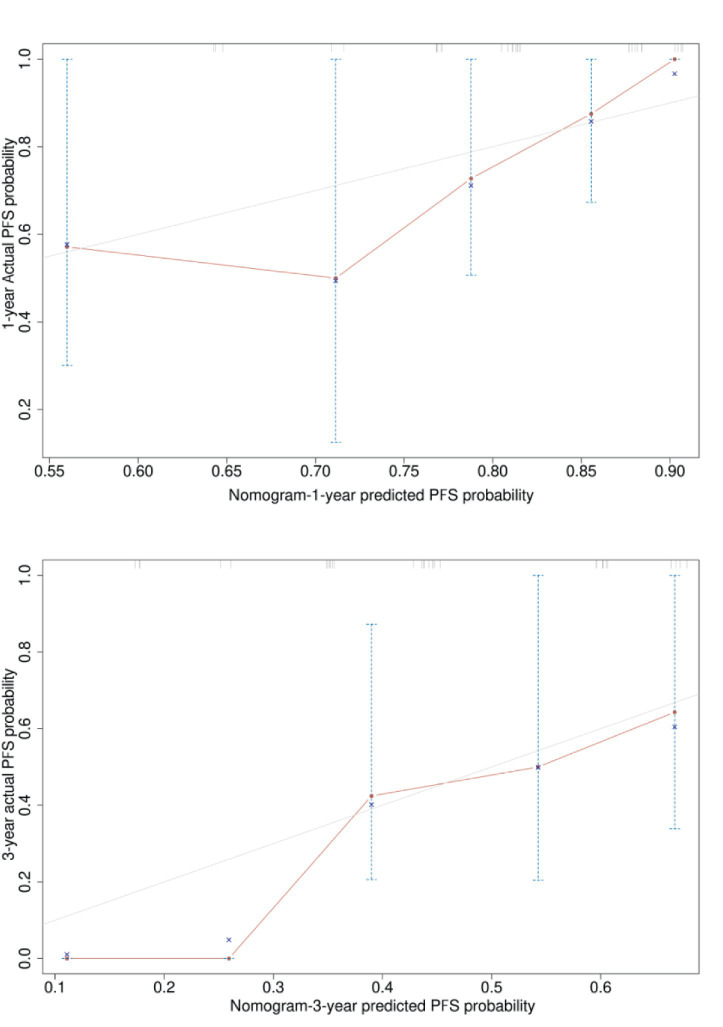
Calibration curve of the PFS rate verification set. [Bootstrap resampling shows that the predicted probabilities of 1-year and 3-year PFS in the validation cohort. The optimism-adjusted C-index after Bootstrap resampling (1,000 repetitions) was 0.69 (95% CI, 0.60–0.78), which corrected the overestimation of the original C-index (0.71, 95% CI, 0.62–0.80) caused by overfitting. This result indicates that the PFS prognostic model also has good discriminative ability in the validation cohort after adjusting for optimism, and the model has good external validity.]

**Figure 4:**
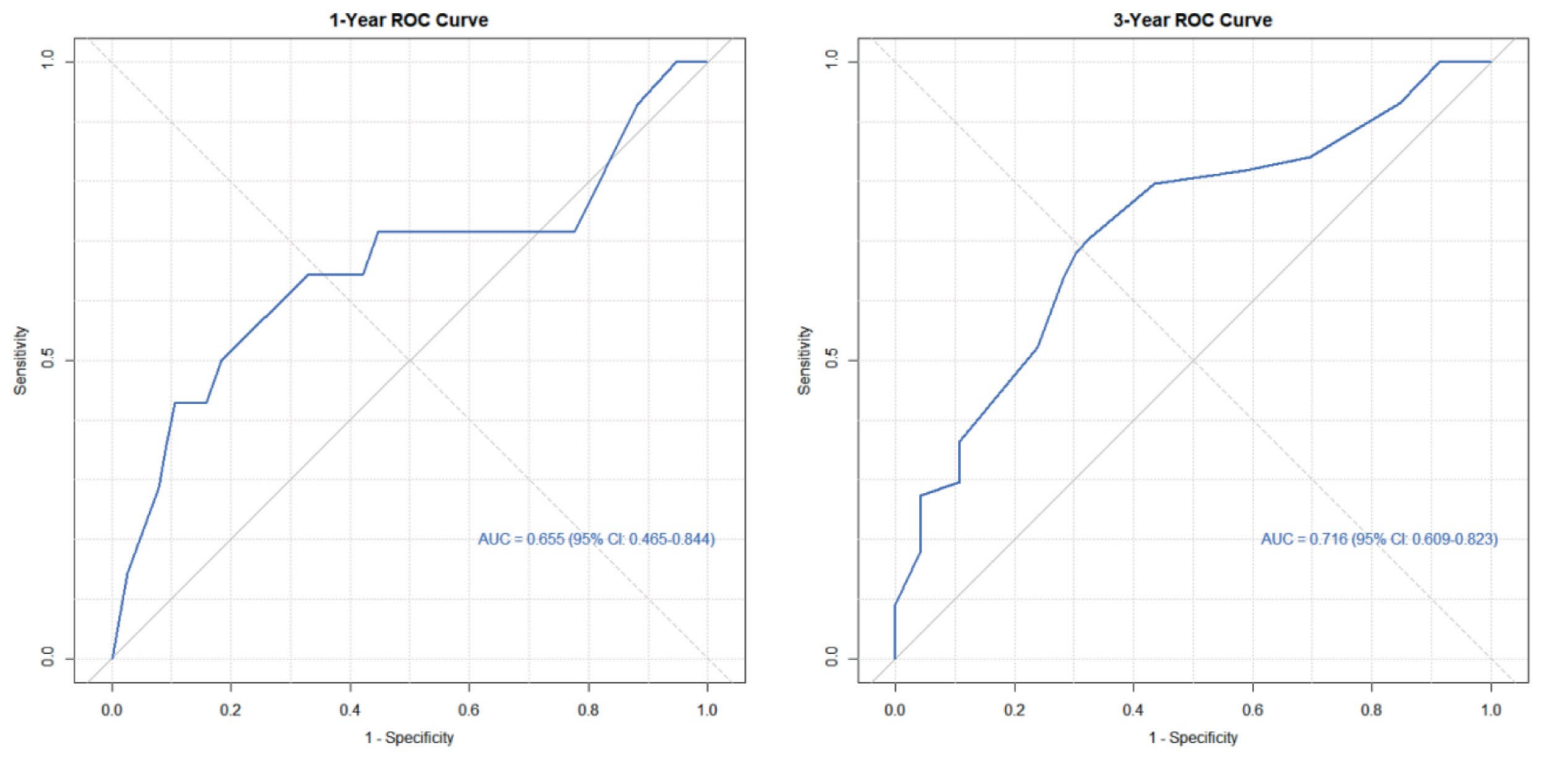
ROC of the PFS rate training cohort. (The results of the time-dependent ROC analysis show that the AUC of the 1-year and 3-year PFS prognostic in the training cohort.)

**Figure 5:**
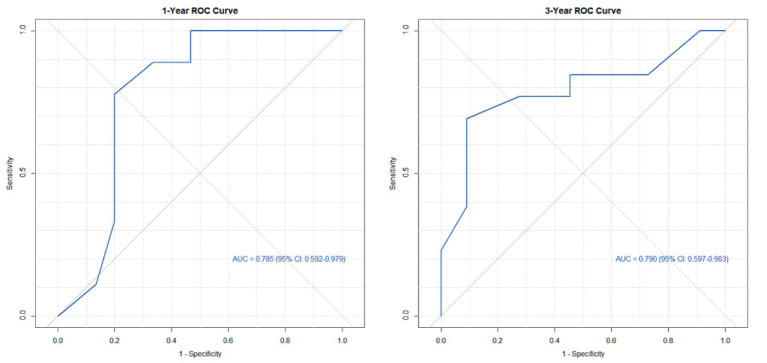
ROC of the PFS rate validation cohort. (The results of the time-dependent ROC analysis show that the AUC of the 1-year and 3-year PFS prognostic in the validation cohort.)

Based on the six risk indicators (brain metastasis, lesion location, maximum tumor diameter, and LMR) screened by the multivariate Cox regression analysis, we constructed a nomogram model (Figure 1) for predicting the 1-year and 3-year PFS rates of EML4-ALK positive lung cancer patients treated with ALK-TKIs. The results of the model performance verification are as follows: (1) Discrimination: The Harrell’s C-index of the training cohort is 0.670, and that of the validation cohort is 0.705, indicating that the model has a stable risk stratification ability. (2) Calibration: Bootstrap resampling (*B* = 500) shows that the predicted probabilities of 1-year and 3-year PFS in both the training cohort and the validation cohort are in good agreement with the actual observed values, as shown in Figures 2, 3. (3) The results of the time-dependent ROC analysis show that the AUC of the 1-year and 3-year PFS prognostic in the training cohort are 0.655 (95% CI: 0.465–0.844) and 0.716 (95% CI: 0.609–0.823), as shown in Figure 4; the AUC of the 1-year and 3-year PFS prognostic in the validation cohort are 0.785 (95% CI: 0.592–0.979) and 0.790 (95% CI: 0.579–0.983), as shown in Figure 5; this further confirms the prognostic accuracy of the model.

#### OS

**Figure 6:**
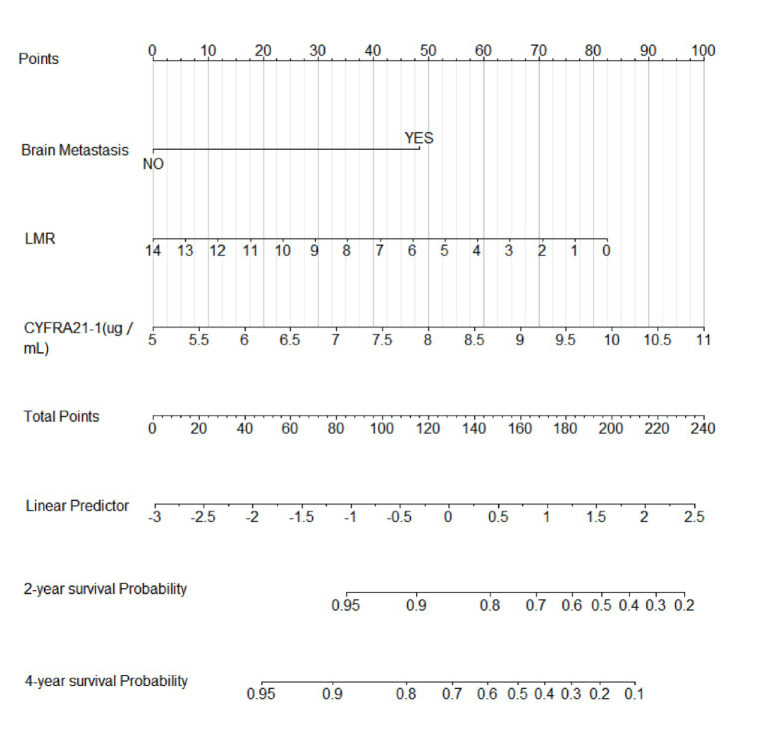
Nomogram model of survival rate. [Based on the three risk indicators (brain metastasis, CYFRA21-1, and LMR) screened by the multivariate Cox regression analysis, a nomogram model for predicting the 2-year and 4-year survival rates of EML4-ALK positive lung cancer patients treated with ALK-TKIs.]

**Figure 7:**
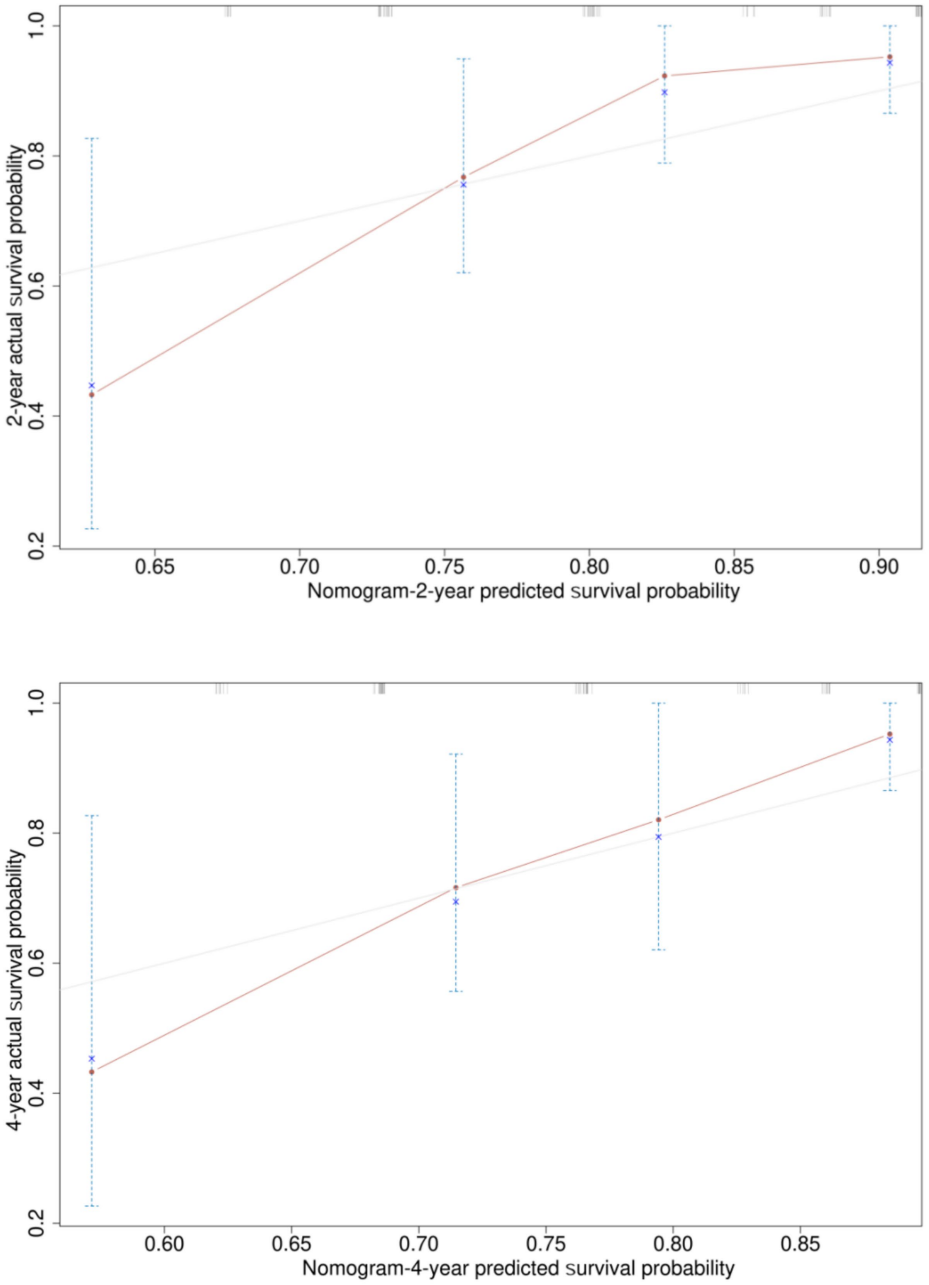
Calibration curve of the survival rate training cohort. [Bootstrap resampling shows that the predicted probabilities of 2-year and 4-year survival in the training cohort. The optimism-adjusted C-index after bootstrap resampling (1,000 repetitions) was 0.72 (95% CI: 0.63–0.81), which corrected the overestimation of the original C-index (0.74, 95% CI: 0.65–0.83) caused by overfitting. This result indicates that the survival prognostic model has good discriminative ability in the training cohort after adjusting for optimism, and the model performance is stable and reliable.]

**Figure 8:**
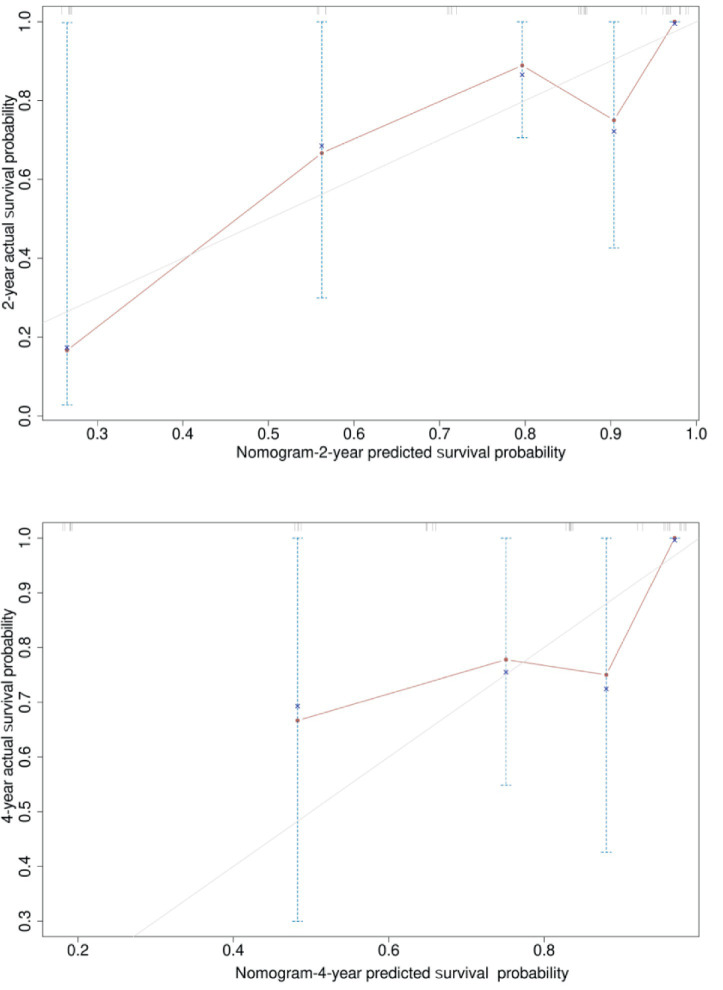
Calibration curve of the survival rate validation cohort. [Bootstrap resampling shows that the predicted probabilities of 2-year and 4-year survival in the validation cohort. The optimism-adjusted C-index after bootstrap resampling (1,000 repetitions) was 0.70 (95% CI: 0.61–0.79), which corrected the overestimation of the original C-index (0.72, 95% CI: 0.63–0.81) caused by overfitting. This result indicates that the survival prognostic model also has good discriminative ability in the validation cohort after adjusting for optimism, and the model has good external validity.]

**Figure 9:**
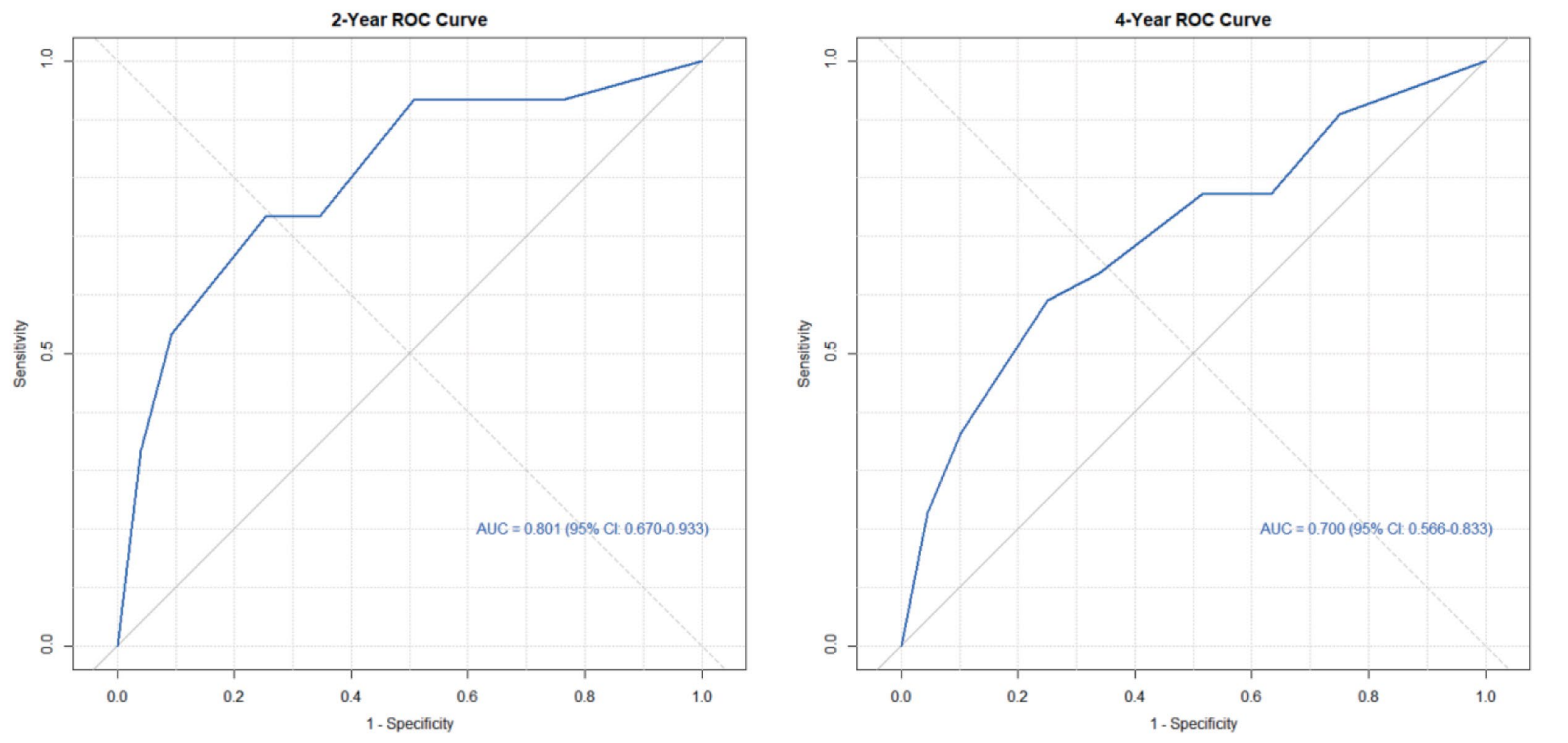
ROC of the survival rate training cohort. (The results of the time-dependent ROC analysis show that the AUC of the 2-year and 4-year survival rate prognostic in the training cohort.)

**Figure 10:**
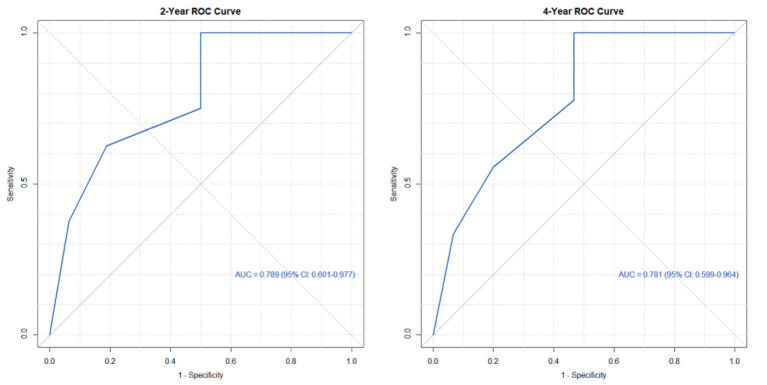
ROC of the survival rate validation cohort. (The results of the time-dependent ROC analysis show that the AUC of the 2-year and 4-year survival rate prognostic in the validation cohort.)

Based on the three risk indicators (brain metastasis, CYFRA21-1, and LMR) screened by the multivariate Cox regression analysis, we constructed a nomogram model (Figure 6) for predicting the 2-year and 4-year survival rates of EML4-ALK positive lung cancer patients treated with ALK-TKIs. The results of the model performance verification are as follows: (1) Discrimination: The Harrell’s C-index of the training cohort is 0.737, and that of the validation cohort is 0.712, indicating that the model has a stable risk stratification ability. (2) Calibration: Bootstrap resampling (*B* = 500) shows that the predicted probabilities of 2-year and 4-year survival in both the training cohort and the validation cohort are in good agreement with the actual observed values, as shown in Figures 7, 8. (3) The results of the time-dependent ROC analysis show that the AUC of the 2-year and 4-year survival rate prognostic in the training cohort are 0.801 (95% CI: 0.670–0.933) and 0.700 (95% CI: 0.566–0.833), as shown in Figure 9; the AUC of the 2-year and 4-year survival rate prognostic in the validation cohort are 0.789 (95% CI: 0.601–0.977) and 0.781 (95% CI: 0.599–0.964), as shown in Figure 10; this further confirms the prognostic accuracy of the model.

## Discussion

Brain metastasis is relatively common among patients with advanced NSCLC. It has been reported that 20 to 30% of NSCLC patients have brain metastasis at the initial diagnosis (8). A multicenter retrospective study (9) involving 4,529 NSCLC patients has pointed out that brain metastasis is closely related to the prognosis of patients, and the greater the number of brain metastases, the worse the prognosis. In patients with advanced ALK-positive NSCLC, some studies have shown that compared with patients without brain metastasis at baseline, the progression-free survival (PFS) of patients with brain metastasis at baseline is shortened (10, 11). This study has also found that brain metastasis at baseline is a risk factor affecting the PFS and OS of EML4-ALK positive NSCLC patients treated with ALK-TKIs, which is consistent with the results of the above reports. This may be closely related to the inhibitory effect of the blood–brain barrier on drug penetration. Most ALK-TKIs have the characteristics of high P-glycoprotein and breast cancer resistance protein substrates, and will be actively cleared by the efflux pumps of the blood–brain barrier, resulting in a cerebrospinal fluid/plasma drug concentration ratio much lower than the required therapeutic threshold (12, 13), which in turn affects the PFS and OS of patients. However, some other studies have drawn different conclusions. In a study by Costa et al. (14), 888 ALK-rearranged NSCLC patients treated with crizotinib in the clinical trials PROFILE1005 and 1,007 were analyzed. Among them, 275 patients (31%) had brain metastases. The results of this study showed that there were no statistically significant differences in disease control rate, objective response rate (ORR), PFS, etc. between patients with and without brain metastases before treatment. Some studies have suggested that the incidence of central lung cancer is higher in ALK-rearranged NSCLC than in ALK-negative NSCLC (15, 16). The results of the multivariate Cox regression analysis in this study showed that the lesion location is an independent influencing factor for the PFS of EML4-ALK positive NSCLC patients treated with ALK-TKIs, and the central type is a risk factor for PFS. In addition, the maximum tumor diameter ≥3 cm is also an independent risk factor affecting the PFS of patients. The study by Choi et al. (17) pointed out that a maximum tumor diameter ≤3 cm and a peripheral lesion location are independent protective factors for the PFS of NSCLC patients treated with TKIs, which is consistent with the results of this study and jointly reveals the important impact of the anatomical characteristics of the tumor on the prognosis of NSCLC patients treated with TKIs. Compared with peripheral NSCLC, central NSCLC has higher whole-genome instability, intratumoral heterogeneity, and tumor stemness index. These factors can make tumor cells more invasive and drug-resistant, affecting the effect of ALK-TKIs treatment, and thus affecting PFS (18). In addition, central NSCLC shows a higher level of tumor evolution (with a higher subclonal diversity index than peripheral NSCLC), which may lead to an increase in the heterogeneity of tumor cells. Tumor cells are more likely to generate drug-resistant mutations, affecting the effect of TKIs treatment and thus affecting PFS (19). As the tumor diameter increases, the hypoxic microenvironment within the tumor tissue intensifies, activating hypoxia-inducible factor (HIF) and other related pathways. This activation promotes epithelial-mesenchymal transition (EMT) in tumor cells, which not only enhances their migratory and invasive abilities but also upregulates the expression of ABC transporters. Consequently, the efflux of targeted drugs from tumor cells increases, leading to drug resistance. Additionally, a larger tumor diameter is accompanied by a more complex angiogenesis network. Abnormal tumor vessel structure and function result in chaotic blood flow and increased vascular permeability. These factors not only impede the effective delivery of drugs to the tumor tissue via the bloodstream but also form a drug penetration barrier, making it difficult for drug concentrations in the tumor core to reach the effective therapeutic threshold. Ultimately, these processes affect the PFS of patients (20, 21).

The LMR, a key indicator reflecting the balance between inflammation and immunity in the body, plays a crucial role in the occurrence and development of tumors (22). Extensive research has demonstrated a significant association between LMR and the prognosis of malignant tumors; patients with higher baseline LMR values tend to have better OS and PFS (23, 24). Our study found that LMR is an independent prognostic factor for both PFS and OS in EML4-ALK-positive NSCLC patients treated with ALK-TKIs, with LMR > 2.26 acting as a protective factor. The underlying mechanism may be related to inflammation-mediated resistance to targeted therapy in EML4-ALK-positive lung cancer. Low LMR is associated with elevated levels of inflammatory cytokines (such as IL-6 and TNF-α) and an inflammatory microenvironment. These cytokines can activate resistance-related pathways, such as MAPK and Wnt/*β*-catenin, thereby attenuating the antitumor effects of ALK-TKIs (25). Additionally, the inflammatory microenvironment can induce overexpression of the ABC transporter family (particularly P-gp/ABCB1), leading to increased efflux of targeted drugs (26). Low LMR (reflecting relative monocytosis) might indicate an increased presence of tumor-associated macrophages (TAMs), which are known to promote tumor progression, angiogenesis, and immunosuppression, thereby contributing to a poorer prognosis. Through multivariate Cox regression analysis, our study confirmed that serum CYFRA21-1 level is an independent prognostic factor for OS in EML4-ALK-positive NSCLC patients. This finding is consistent with previous studies (27, 28), which reported that high CYFRA21-1 levels are significantly associated with reduced efficacy of targeted therapy in NSCLC patients, possibly reflecting increased tumor burden and enhanced treatment resistance.

A simple and practical prognostic model is beneficial for guiding medical staff in formulating relevant measures. The nomogram is a commonly used prognostic model in clinical practice, which can predict the survival rate of tumor patients to a certain extent. In this study, based on the independent influencing factors of PFS and OS screened by multivariate Cox regression, we constructed nomogram models for predicting PFS and OS respectively, and verified them. The results showed that in the prognostic of PFS rate, the AUC of the 1-year and 3-year PFS rate prognostic in the training cohort were 0.655 and 0.716, respectively, and the AUC of the 1-year and 3-year PFS prognostic in the validation cohort were 0.785 and 0.790, respectively; the AUC values of both the training cohort and the validation cohort were greater than 0.6, indicating that the model has reliable prognostic accuracy for short-term (1-year) and long-term (3-year) PFS. It is worth noting that as the prognostic time is extended, the discrimination efficiency of the training cohort shows an increasing trend (ΔAUC = +0.061), suggesting that the model has better prognostic stability for long-term prognosis. In the prognostic of OS rate, the AUC of the 2-year and 4-year OS rate prognostic in the training cohort were 0.801 and 0.700 respectively, and the AUC of the 2-year and 4-year OS rate prognostic in the validation cohort were 0.789 and 0.781, respectively. The AUC values of both the training cohort and the validation cohort were above 0.7, indicating that the model has a good prognostic effect on the overall survival of patients and can distinguish the survival prognosis of different patients to a certain extent. In addition, the AUC values of the 2-year and 4-year OS rate prognostic in the validation cohort were similar to those in the training cohort, indicating that the model has a certain degree of stability in OS prognostic and performs consistently across different datasets. At the same time, in this study, the prognostic model was internally validated by the Bootstrap resampling method (*B* = 500), and the results showed that the predicted probabilities of 1-year and 3-year PFS, as well as 2-year and 4-year OS of the model were in good agreement with the actual values. In summary, the OS/PFS prognostic model constructed in this study demonstrates reliable prognostic efficiency and can provide an individualized prognostic assessment tool for clinicians.

However, this study also has limitations. This single-center, single-arm retrospective study has several limitations: it only collected data from one hospital with a relatively small sample size, leading to limited representativeness and a risk of model overfitting; follow-up duration was insufficient, resulting in immature overall survival (OS) data and a limited number of events; molecular detection and analysis of acquired resistance mutations were not performed in patients with post-treatment progression; efficacy differences among various ALK-TKIs and treatment-covariate interactions were not evaluated, making the established prognostic model only applicable to first- and second-generation ALK-TKI-treated patients (not lorlatinib or other latest-generation inhibitors) and unable to guide treatment regimen selection; the model was not statistically compared head-to-head with existing well-established scoring systems (e.g., ALK-BPI, Lung-molGPA); and the multivariate model did not fully adjust for confounding factors such as central nervous system (CNS) disease burden, CNS-directed local therapy, TKI selection, indication bias, and treatment lines, with insufficient detailed CNS-related data limiting causal interpretation of brain metastasis prognostic impact.

Future studies should adopt prospective, multi-center cohort designs with larger sample sizes, enroll patients treated with different ALK-TKIs (including third-generation agents), evaluate treatment-covariate interactions, control for confounding factors, and collect comprehensive CNS-related data. Additionally, extended follow-up is needed for more mature OS endpoints; prespecified cutoff values or retained continuous variables should be used to reduce overfitting and improve external validity; head-to-head comparisons with established scoring systems should be conducted; and external validation in larger, independent, prospective cohorts is required before clinical recommendation of the model.

This study has several innovations. Most existing models focus on clinical characteristics and metastatic status, and few simultaneously incorporate systemic inflammatory markers (LMR, PNI, NLR) and tumor lesion features. Based on real-world clinical data, this study constructed a simple and practical prognostic model with readily available variables, facilitating clinical application and popularization. The model was internally validated using a 70/30 data split and evaluated with the Bootstrap-corrected optimism-adjusted C-index, which improved the model’s robustness and clinical applicability.

The validation cohort in this study was regarded as an internal validation subgroup rather than an independent external cohort. In conclusion, brain metastasis, lesion location, maximum tumor diameter, and LMR are factors influencing the PFS of EML4-ALK positive NSCLC patients treated with ALK-TKIs. Brain metastasis, the level of CYFRA21-1, and LMR are related to the OS. The prognostic models for PFS and OS constructed based on these factors have certain clinical prognostic value.

## Data Availability

The original contributions presented in the study are included in the article/supplementary material, further inquiries can be directed to the corresponding author.
